# The genome sequence of a ground beetle, *Pterostichus niger *(Schaller, 1783)

**DOI:** 10.12688/wellcomeopenres.20418.1

**Published:** 2023-11-23

**Authors:** Maxwell V. L. Barclay, Michael Geiser, Danaë Vassiliades, Will Bayfield Farrell, Joana Cristóvão, Natural History Museum Genome Acquisition Lab, Darwin Tree of Life Barcoding collective, Wellcome Sanger Institute Tree of Life Management, Samples and Laboratory team, Wellcome Sanger Institute Scientific Operations: Sequencing Operations, Wellcome Sanger Institute Tree of Life Core Informatics team, Tree of Life Core Informatics collective, Darwin Tree of Life Consortium

**Affiliations:** 1 The Marine Biological Association, Plymouth, England, UK

**Keywords:** Pterostichus niger, ground beetle, genome sequence, chromosomal, Coleoptera

## Abstract

We present a genome assembly from an individual male *Pterostichus niger* (a ground beetle; Arthropoda; Insecta; Coleoptera; Carabidae). The genome sequence is 674.1 megabases in span. Most of the assembly is scaffolded into 19 chromosomal pseudomolecules, including the X sex chromosome. The mitochondrial genome has also been assembled and is 17.16 kilobases in length.

## Species taxonomy

Eukaryota; Metazoa; Eumetazoa; Bilateria; Protostomia; Ecdysozoa; Panarthropoda; Arthropoda; Mandibulata; Pancrustacea; Hexapoda; Insecta; Dicondylia; Pterygota; Neoptera; Endopterygota; Coleoptera; Adephaga; Caraboidea; Carabidae; Harpalinae; Pterostichini; *Pterostichus*; *Platysma*; *Pterostichus niger* (Schaller, 1783) (NCBI:txid106386).

## Background

The ground beetle *Pterostichus niger* (Schaller, 1783) is the largest of 18 species of the genus *Pterostichus Bonelli, 1810* occurring in Britain and Ireland, and the only British member of the subgenus *Platysma Bonelli, 1810*. It is common and generally distributed throughout the British Isles, including in the Orkneys, the Shetlands, and most of the smaller islands. Like many larger Carabidae in Britain, adults and larvae are terrestrial predators, and *P. niger* can be abundant in a range of habitats from forests to moorlands and mountains, usually favouring damper biotopes. Globally, the Palaearctic Catalogue lists *P. niger* for 39 European and 7 Asian countries, most frequently in the north but extending southwest to Spain and east to West Siberia and Western China, but it is apparently absent from Portugal and North Africa ( Löbl & Löbl, 2017).

*Pterostichus niger* is a distinctive insect, and in Britain could only be confused with the similarly sized carabids *Pterostichus melanarius* (Illiger, 1798) and *Abax parallelepipedus* (Piller & Mitterpacher, 1783), both of which can be abundant in the same habitats, but which can be easily differentiated by the shape of the pronotum. Species identification is straightforward using Luff (2007) or Lindroth (1974). *P. niger* is a nocturnal predator primarily of slugs, worms and insect larvae on the ground and in forest litter. Adults can be collected almost all year, by night-searching, looking under logs and loose bark, or by pitfall trapping. It has flight wings, but it appears that most individuals do not have the flight muscles developed and the species is rarely if ever observed to fly.

The genome of *Pterostichus niger* was sequenced as part of the Darwin Tree of Life Project, a collaborative effort to sequence all named eukaryotic species in the Atlantic Archipelago of Britain and Ireland. Here we present a chromosomally complete genome sequence for *Pterostichus niger*, based on one male specimen collected in September 2021 by a small Natural History Museum team at Bookham Common, Surrey, England.

## Genome sequence report

**Figure 1.**
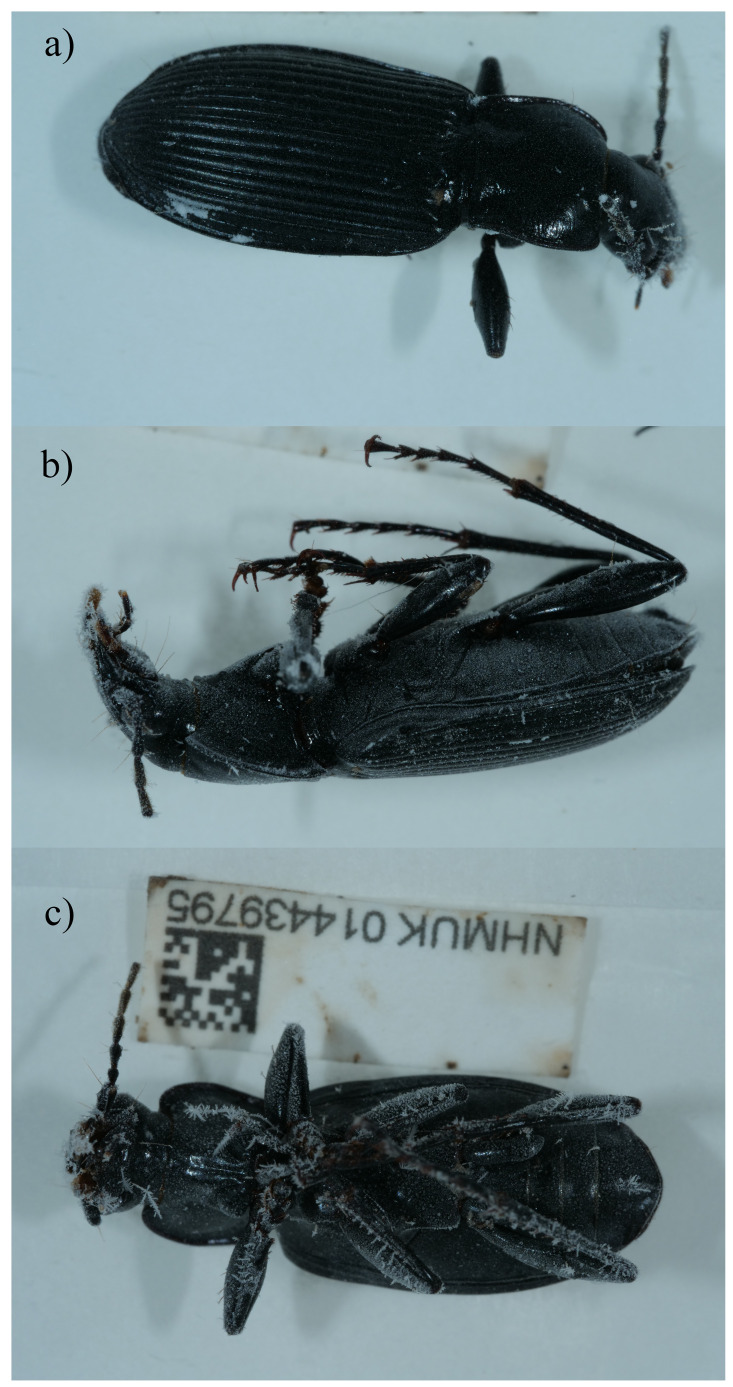
Photograph of the *Pterostichus niger* (icPteNige1) specimen used for genome sequencing. **a**) Dorsal view, **b**) Lateral view and **c**) Ventral view.

The genome was sequenced from one male *Pterostichus niger* ( Figure 1) collected from Bookham Common, England, UK (51.29, –0.39, Ordnance Survey Grid Reference TQ1256). A total of 29-fold coverage in Pacific Biosciences single-molecule HiFi long reads was generated. Primary assembly contigs were scaffolded with chromosome conformation Hi-C data. Manual assembly curation corrected 175 missing joins or mis-joins and removed 10 haplotypic duplications, reducing the assembly length by 0.75% and the scaffold number by 40.92%, and increasing the scaffold N50 by 18.2%.

**Figure 2.**
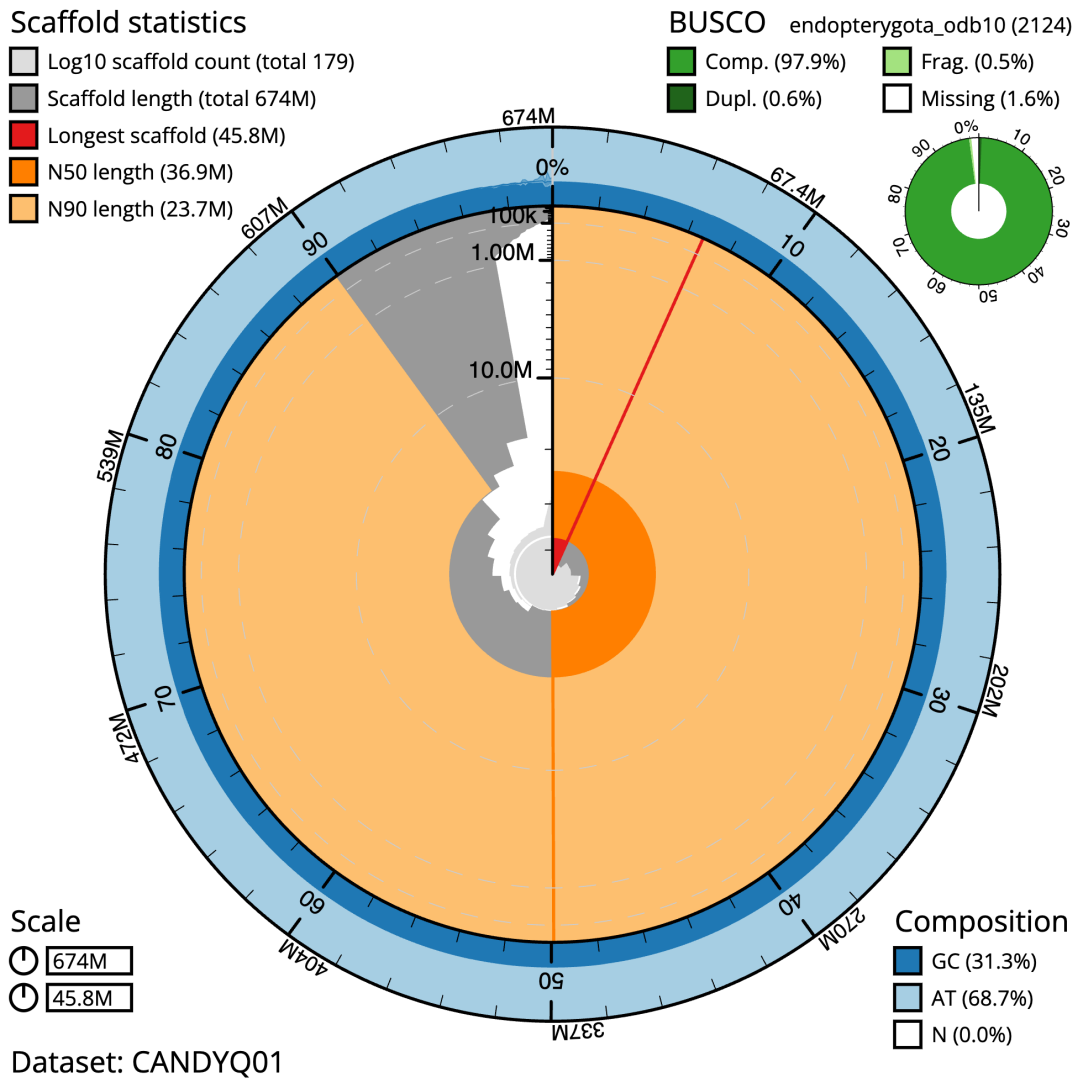
Genome assembly of *Pterostichus niger*, icPteNige1.1: metrics. The BlobToolKit Snailplot shows N50 metrics and BUSCO gene completeness. The main plot is divided into 1,000 size-ordered bins around the circumference with each bin representing 0.1% of the 674,098,515 bp assembly. The distribution of scaffold lengths is shown in dark grey with the plot radius scaled to the longest scaffold present in the assembly (45,804,315 bp, shown in red). Orange and pale-orange arcs show the N50 and N90 scaffold lengths (36,870,366 and 23,707,136 bp), respectively. The pale grey spiral shows the cumulative scaffold count on a log scale with white scale lines showing successive orders of magnitude. The blue and pale-blue area around the outside of the plot shows the distribution of GC, AT and N percentages in the same bins as the inner plot. A summary of complete, fragmented, duplicated and missing BUSCO genes in the endopterygota_odb10 set is shown in the top right. An interactive version of this figure is available at https://blobtoolkit.genomehubs.org/view/icPteNige1.1/dataset/CANDYQ01/snail.

**Figure 3.**
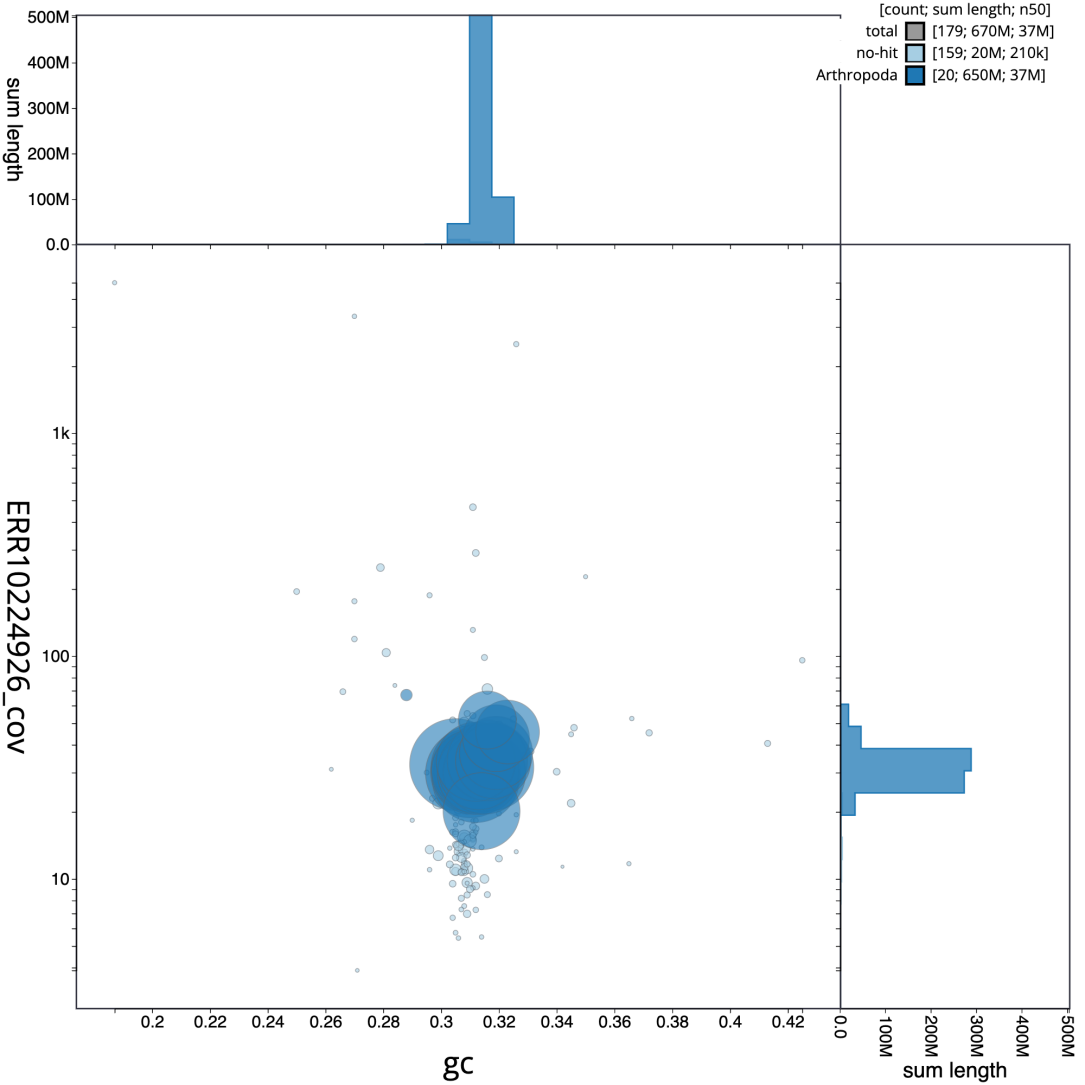
Genome assembly of *Pterostichus niger*, icPteNige1.1: BlobToolKit GC-coverage plot. Scaffolds are coloured by phylum. Circles are sized in proportion to scaffold length. Histograms show the distribution of scaffold length sum along each axis. An interactive version of this figure is available at https://blobtoolkit.genomehubs.org/view/icPteNige1.1/dataset/CANDYQ01/blob.

**Figure 4.**
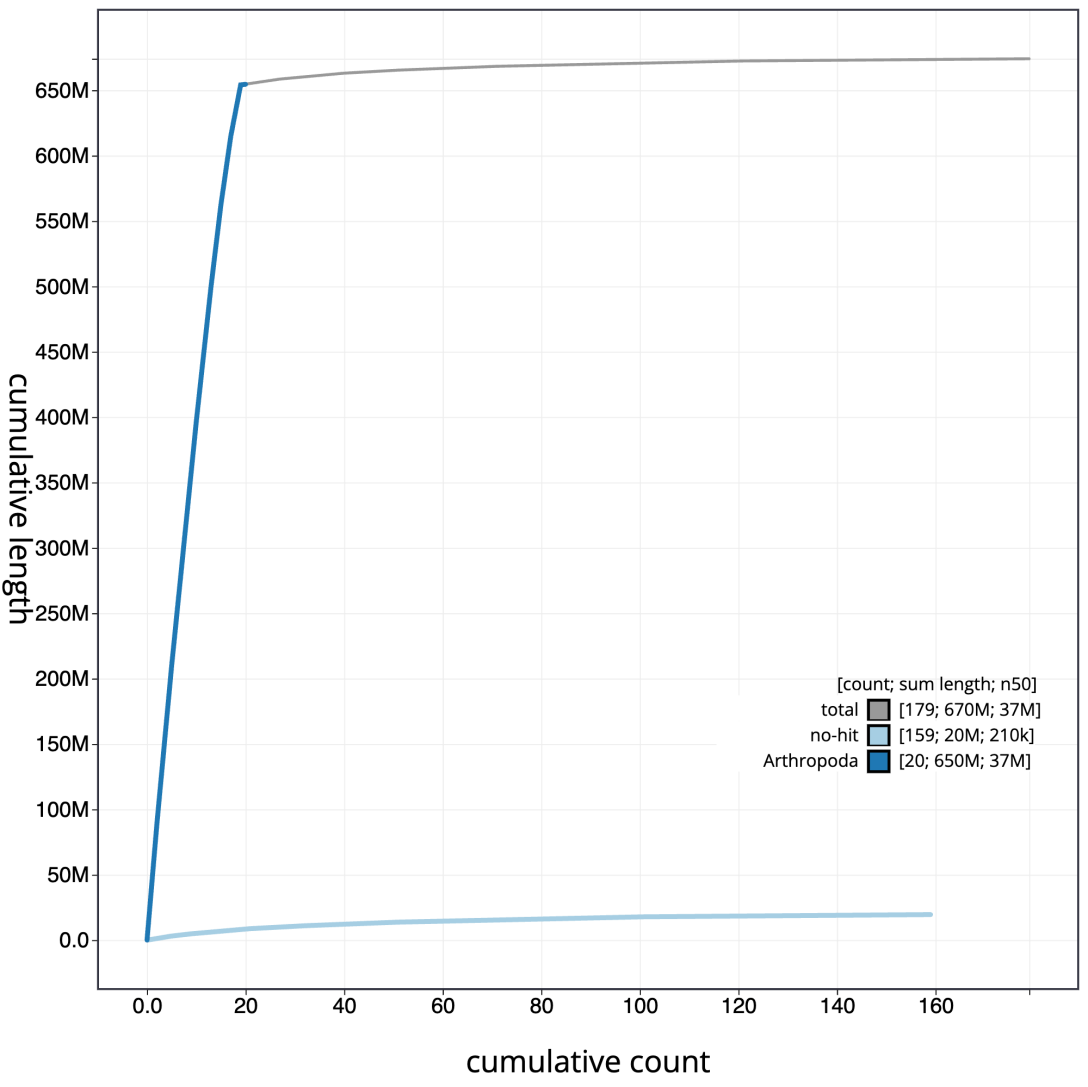
Genome assembly of *Pterostichus niger*, icPteNige1.1: BlobToolKit cumulative sequence plot. The grey line shows cumulative length for all scaffolds. Coloured lines show cumulative lengths of scaffolds assigned to each phylum using the buscogenes taxrule. An interactive version of this figure is available at https://blobtoolkit.genomehubs.org/view/icPteNige1.1/dataset/CANDYQ01/cumulative.

**Figure 5.**
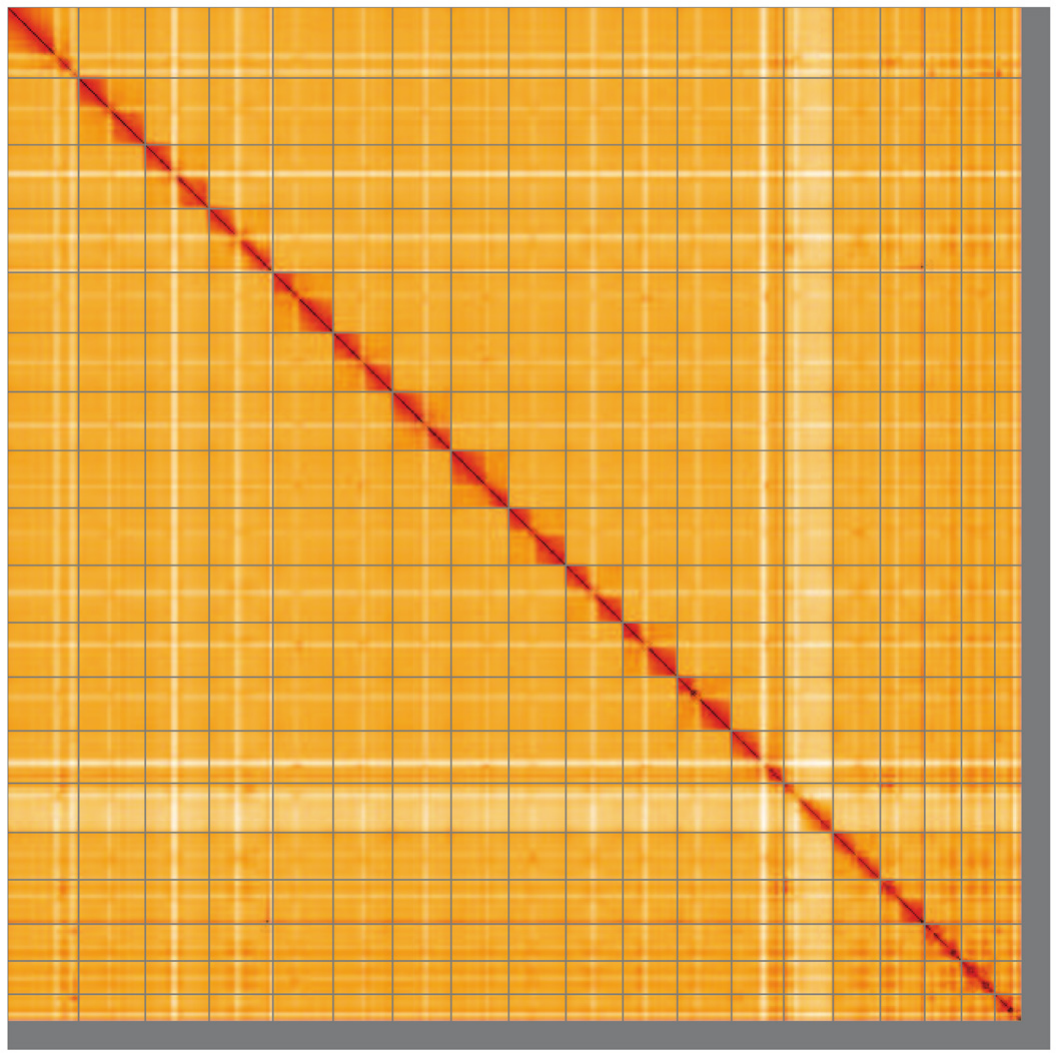
Genome assembly of *Pterostichus niger*, icPteNige1.1: Hi-C contact map of the icPteNige1.1 assembly, visualised using HiGlass. Chromosomes are shown in order of size from left to right and top to bottom. An interactive version of this figure may be viewed at https://genome-note-higlass.tol.sanger.ac.uk/l/?d=V0B2AOZYSaae37-GJDYNVg.

The final assembly has a total length of 674.1 Mb in 178 sequence scaffolds with a scaffold N50 of 36.9 Mb ( Table 1). The snailplot in Figure 2 provides a summary of the assembly statistics, while the distribution of assembly scaffolds on GC proportion and coverage is shown in Figure 3. The cumulative assembly plot in Figure 4 shows curves for subsets of scaffolds assigned to different phyla. Most (97.06%) of the assembly sequence was assigned to 19 chromosomal-level scaffolds, representing 18 autosomes and the X sex chromosome. Chromosome-scale scaffolds confirmed by the Hi-C data are named in order of size ( Figure 5; Table 2). While not fully phased, the assembly deposited is of one haplotype. Contigs corresponding to the second haplotype have also been deposited. The mitochondrial genome was also assembled and can be found as a contig within the multifasta file of the genome submission.

**Table 1.** Genome data for *Pterostichus niger*, icPteNige1.1.

Project accession data		
Assembly identifier	icPteNige1.1	
Assembly release date	2022-12-04	
Species	*Pterostichus niger*	
Specimen	icPteNige1	
NCBI taxonomy ID	106386	
BioProject	PRJEB56056	
BioSample ID	SAMEA14448385	
Isolate information	icPteNige1, male: thorax (DNA sequencing), head (Hi-C data), abdomen (RNA sequencing)	
Assembly metrics *		*Benchmark*
Consensus quality (QV)	61.2	*≥ 50*
*k*-mer completeness	100%	*≥ 95%*
BUSCO **	C:97.9%[S:97.3%,D:0.6%],F:0.5%,M :1.6%,n:2,124	*C ≥ 95%*
Percentage of assembly mapped to chromosomes	97.06%	*≥ 95%*
Sex chromosomes	X chromosome	*localised homologous pairs*
Organelles	Mitochondrial genome assembled	*complete single alleles*
Raw data accessions		
PacificBiosciences SEQUEL II	ERR10224926	
Hi-C Illumina	ERR10297817	
PolyA RNA-Seq Illumina	ERR11641105	
Genome assembly		
Assembly accession	GCA_947425015.1	
*Accession of alternate haplotype*	GCA_947425025.1	
Span (Mb)	674.1	
Number of contigs	596	
Contig N50 length (Mb)	2.8	
Number of scaffolds	178	
Scaffold N50 length (Mb)	36.9	
Longest scaffold (Mb)	45.8	

* Assembly metric benchmarks are adapted from column VGP-2020 of “Table 1: Proposed standards and metrics for defining genome assembly quality” from Rhie *et al.* (2021). ** BUSCO scores based on the endopterygota_odb10 BUSCO set using v5.3.2. C = complete [S = single copy, D = duplicated], F = fragmented, M = missing, n = number of orthologues in comparison. A full set of BUSCO scores is available at https://blobtoolkit.genomehubs.org/view/icPteNige1.1/dataset/CANDYQ01/busco.

**Table 2.** Chromosomal pseudomolecules in the genome assembly of Pterostichus niger, icPteNige1.

INSDC accession	Chromosome	Length (Mb)	GC%
OX380334.1	1	45.8	30.5
OX380335.1	2	43.02	31.0
OX380336.1	3	41.22	31.0
OX380337.1	4	41.09	31.5
OX380338.1	5	38.84	31.0
OX380339.1	6	38.2	31.0
OX380340.1	7	37.79	31.0
OX380341.1	8	37.09	31.0
OX380342.1	9	36.87	31.5
OX380343.1	10	36.79	31.5
OX380344.1	11	35.15	31.5
OX380345.1	12	34.8	31.5
OX380346.1	13	33.68	31.0
OX380348.1	14	30.38	32.0
OX380349.1	15	28.65	32.0
OX380350.1	16	23.71	32.0
OX380351.1	17	21.51	32.5
OX380352.1	18	17.66	31.5
OX380347.1	X	31.87	31.5
OX380353.1	MT	0.02	19.0

The estimated Quality Value (QV) of the final assembly is 61.2 with *k*-mer completeness of 100%, and the assembly has a BUSCO v5.3.2 completeness of 97.9% (single = 97.3%, duplicated = 0.6%), using the endopterygota_odb10 reference set ( *n* = 2,124).

Metadata for specimens, barcode results, spectra estimates, sequencing runs, contaminants and pre-curation assembly statistics are given at https://links.tol.sanger.ac.uk/species/106386.

## Methods

### Sample acquisition and nucleic acid extraction

A male *Pterostichus niger* (specimen ID NHMUK014439795, ToLID icPteNige1) was collected from Bookham Common, England, UK (latitude 51.29, longitude –0.39) on 2021-09-19. The specimen was collected by Maxwell Barclay, Michael Geiser, Danaë Vassiliades, Will Bayfield Farrell and Joana Cristóvão and identified by Maxwell Barclay (Natural History Museum). The specimen was dry frozen at –80 °C.

The workflow for high molecular weight (HMW) DNA extraction at the Wellcome Sanger Institute (WSI) includes a sequence of core procedures: sample preparation; sample homogenisation; DNA extraction; HMW DNA fragmentation; and fragmented DNA clean-up. The icPteNige1 sample was prepared for DNA extraction at the WSI Tree of Life laboratory: it was weighed and dissected on dry ice with tissue set aside for Hi-C sequencing ( https://dx.doi.org/10.17504/protocols.io.x54v9prmqg3e/v1). Sample homogenisation was carried on using the Powermasher protocol ( https://dx.doi.org/10.17504/protocols.io.5qpvo3r19v4o/v1). DNA was extracted at the WSI Scientific Operations core using the Qiagen MagAttract HMW DNA kit, according to the manufacturer’s instructions.

RNA was extracted from abdomen tissue of icPteNige1 using the Automated MagMax™ mirVana protocol ( https://dx.doi.org/10.17504/protocols.io.6qpvr36n3vmk/v1). The RNA concentration was assessed using a Nanodrop spectrophotometer and Qubit Fluorometer using the Qubit RNA Broad-Range (BR) Assay kit. Analysis of the integrity of the RNA was done using the Agilent RNA 6000 Pico Kit and Eukaryotic Total RNA assay.

Protocols employed by the Tree of Life laboratory are publicly available on protocols.io: https://dx.doi.org/10.17504/protocols.io.8epv5xxy6g1b/v1.

### Sequencing

Pacific Biosciences HiFi circular consensus DNA sequencing libraries were constructed according to the manufacturers’ instructions. Poly(A) RNA-Seq libraries were constructed using the NEB Ultra II RNA Library Prep kit. DNA and RNA sequencing was performed by the Scientific Operations core at the WSI on Pacific Biosciences SEQUEL II (HiFi) and Illumina NovaSeq 6000 (RNA-Seq) instruments. Hi-C data were also generated from head tissue of icPteNige1 using the Arima2 kit and sequenced on the Illumina NovaSeq 6000 instrument.

### Genome assembly, curation and evaluation

Assembly was carried out with Hifiasm ( Cheng *et al.*, 2021) and haplotypic duplication was identified and removed with purge_dups ( Guan *et al.*, 2020). The assembly was then scaffolded with Hi-C data ( Rao *et al.*, 2014) using YaHS ( Zhou *et al.*, 2023). The assembly was checked for contamination and corrected using the gEVAL system ( Chow *et al.*, 2016) as described previously ( Howe *et al.*, 2021). Manual curation was performed using gEVAL, HiGlass ( Kerpedjiev *et al.*, 2018) and Pretext ( Harry, 2022). The mitochondrial genome was assembled using MitoHiFi ( Uliano-Silva *et al.*, 2023), which runs MitoFinder ( Allio *et al.*, 2020) or MITOS ( Bernt *et al.*, 2013) and uses these annotations to select the final mitochondrial contig and to ensure the general quality of the sequence.

A Hi-C map for the final assembly was produced using bwa-mem2 ( Vasimuddin *et al.*, 2019) in the Cooler file format ( Abdennur & Mirny, 2020). To assess the assembly metrics, the *k*-mer completeness and QV consensus quality values were calculated in Merqury ( Rhie *et al.*, 2020). This work was done using Nextflow ( Di Tommaso *et al.*, 2017) DSL2 pipelines “sanger-tol/readmapping” ( Surana *et al.*, 2023a) and “sanger-tol/genomenote” ( Surana *et al.*, 2023b). The genome was analysed within the BlobToolKit environment ( Challis *et al.*, 2020) and BUSCO scores ( Manni *et al.*, 2021; Simão *et al.*, 2015) were calculated.

Table 3 contains a list of relevant software tool versions and sources.

**Table 3.** Software tools: versions and sources.

Software tool	Version	Source
BlobToolKit	4.1.5	https://github.com/blobtoolkit/blobtoolkit
BUSCO	5.3.2	https://gitlab.com/ezlab/busco
Hifiasm	0.16.1-r375	https://github.com/chhylp123/hifiasm
HiGlass	1.11.6	https://github.com/higlass/higlass
Merqury	MerquryFK	https://github.com/thegenemyers/MERQURY.FK
MitoHiFi	2	https://github.com/marcelauliano/MitoHiFi
PretextView	0.2	https://github.com/wtsi-hpag/PretextView
purge_dups	1.2.3	https://github.com/dfguan/purge_dups
sanger-tol/genomenote	v1.0	https://github.com/sanger-tol/genomenote
sanger-tol/readmapping	1.1.0	https://github.com/sanger-tol/readmapping/tree/1.1.0
YaHS	yahs-1.1.91eebc2	https://github.com/c-zhou/yahs

### Wellcome Sanger Institute – Legal and Governance

The materials that have contributed to this genome note have been supplied by a Darwin Tree of Life Partner. The submission of materials by a Darwin Tree of Life Partner is subject to the **‘Darwin Tree of Life Project Sampling Code of Practice’**, which can be found in full on the Darwin Tree of Life website here. By agreeing with and signing up to the Sampling Code of Practice, the Darwin Tree of Life Partner agrees they will meet the legal and ethical requirements and standards set out within this document in respect of all samples acquired for, and supplied to, the Darwin Tree of Life Project.

Further, the Wellcome Sanger Institute employs a process whereby due diligence is carried out proportionate to the nature of the materials themselves, and the circumstances under which they have been/are to be collected and provided for use. The purpose of this is to address and mitigate any potential legal and/or ethical implications of receipt and use of the materials as part of the research project, and to ensure that in doing so we align with best practice wherever possible. The overarching areas of consideration are:

•   Ethical review of provenance and sourcing of the material

•   Legality of collection, transfer and use (national and international)

Each transfer of samples is further undertaken according to a Research Collaboration Agreement or Material Transfer Agreement entered into by the Darwin Tree of Life Partner, Genome Research Limited (operating as the Wellcome Sanger Institute), and in some circumstances other Darwin Tree of Life collaborators.

## Data Availability

European Nucleotide Archive: *Pterostichus niger*. Accession number PRJEB56056; https://identifiers.org/ena.embl/PRJEB56056 ( Wellcome Sanger Institute, 2022). The genome sequence is released openly for reuse. The *Pterostichus niger* genome sequencing initiative is part of the Darwin Tree of Life (DToL) project. All raw sequence data and the assembly have been deposited in INSDC databases. The genome will be annotated using available RNA-Seq data and presented through the Ensembl pipeline at the European Bioinformatics Institute. Raw data and assembly accession identifiers are reported in Table 1.

## Author information

Members of the Natural History Museum Genome Acquisition Lab are listed here: https://doi.org/10.5281/zenodo.7139035.

Members of the Darwin Tree of Life Barcoding collective are listed here: https://doi.org/10.5281/zenodo.4893703.

Members of the Wellcome Sanger Institute Tree of Life Management, Samples and Laboratory Team are listed here: https://doi.org/10.5281/zenodo.10066175.

Members of Wellcome Sanger Institute Scientific Operations: Sequencing Operations are listed here: https://doi.org/10.5281/zenodo.10043364.

Members of the Wellcome Sanger Institute Tree of Life Core Informatics team are listed here: https://doi.org/10.5281/zenodo.10066637.

Members of the Tree of Life Core Informatics collective are listed here: https://doi.org/10.5281/zenodo.5013541.

Members of the Darwin Tree of Life Consortium are listed here: https://doi.org/10.5281/zenodo.4783558.
