# Supplementary material for: The genome sequence of a ground beetle, Pterostichus niger (Schaller, 1783)
Source: Wellcome Open Res. 2023 Nov 23;8:544. [Version 1] doi: 10.12688/wellcomeopenres.20418.1 (PMC10907878; doi:10.12688/wellcomeopenres.20418.1)
Supplement: Peer review history

# Reviewer report: Reviewer response for version 1

**DOI:** 10.21956/wellcomeopenres.22607.r74327

**Published:** 2024-03-01

**Authors:** Sudhakar Sivasubramaniam, Arun Arumugaperumal

**Affiliations:** 1 Manonmaniam Sundaranar University, Tirunelveli, Tamil Nadu, India; 2 Department of Biotechnology, Rajalakshmi Engineering College, Chennai, Tamilnadu, India

The authors have sequenced the whole genome of the ground beetle  Pterostichus niger. This is the first article reporting the genome of the species. The genome size was reported as 674.1 Mbp with 19 chromosomes and a mitochondrial genome of 17.6 kbp. Figure 1 shows the specimen photograph of the insect. It would have been better to place a scale and record the photograph to know the dimensions better. There is an id 'icPteNige1' in the figure caption. Authors can mention which record or database that id refers to. While referring to the genus as Pterostichus Bonelli, the word 'Bonelli' should not be italicized as it is not a species name. The provided metadata link https://links.tol.sanger.ac.uk/species/106386 was not working. The authors need to check whether the link is correctly printed. Was the insect tissue cleaned before dissection? Is the entire body of the insect used for DNA extraction or a specific organ/tissue?

Are sufficient details of methods and materials provided to allow replication by others?

Yes

Is the rationale for creating the dataset(s) clearly described?

Yes

Are the datasets clearly presented in a useable and accessible format?

Yes

Are the protocols appropriate and is the work technically sound?

Yes

Reviewer Expertise:

Genomics, Transcriptomics, Regeneration biology

We confirm that we have read this submission and believe that we have an appropriate level of expertise to confirm that it is of an acceptable scientific standard.

# Reviewer report: Reviewer response for version 1

**DOI:** 10.21956/wellcomeopenres.22607.r74325

**Published:** 2024-02-21

**Authors:** Yoshiaki Morino

**Affiliations:** 1 Institute of Life and Environmental Sciences, University of Tsukuba, Tsukuba, Japan

This paper reports the genome sequence of the ground beetle Pterostichus niger. The genome assembly size was 674.1 Mbp, and most of the sequence was assigned to 19 chromosome-level scaffolds. The BUSCO gene completeness score was 97.9%. The quality of this assembly met the standards of the Darwin Tree of Life Project. Therefore, this assembly will be a valuable resource for diverse fields such as entomology, ecotoxicology, and population ecology.

Are sufficient details of methods and materials provided to allow replication by others?

Yes

Is the rationale for creating the dataset(s) clearly described?

Yes

Are the datasets clearly presented in a useable and accessible format?

Yes

Are the protocols appropriate and is the work technically sound?

Yes

Reviewer Expertise:

Marine invertebrate, Evo-Devo

I confirm that I have read this submission and believe that I have an appropriate level of expertise to confirm that it is of an acceptable scientific standard.
